# Challenges and advances in glioblastoma targeted therapy: the promise of drug repurposing and biomarker exploration

**DOI:** 10.3389/fonc.2024.1441460

**Published:** 2024-10-08

**Authors:** William Han Bae, Stefania Maraka, Ahmad Daher

**Affiliations:** ^1^ Division of Hematology/Oncology, Department of Internal Medicine, University of Illinois Chicago, Chicago, IL, United States; ^2^ Department of Neurology and Rehabilitation, University of Illinois Chicago, Chicago, IL, United States

**Keywords:** glioblastoma, targeted therapy, drug repurposing, liquid biopsy, extracellular vesicle (EV), ctDNA

## Abstract

Glioblastoma remains the most prevalent and aggressive primary malignant brain tumor in adults, characterized by limited treatment options and a poor prognosis. Previous drug repurposing efforts have yielded only marginal survival benefits, particularly those involving inhibitors targeting receptor tyrosine kinase and cyclin-dependent kinase-retinoblastoma pathways. This limited efficacy is likely due to several critical challenges, including the tumor’s molecular heterogeneity, the dynamic evolution of its genetic profile, and the restrictive nature of the blood-brain barrier that impedes effective drug delivery. Emerging diagnostic tools, such as circulating tumor DNA and extracellular vesicles, offer promising non-invasive methods for real-time tumor monitoring, potentially enabling the application of targeted therapies to more selected patient populations. Moreover, innovative drug delivery strategies, including focused ultrasound, implantable drug-delivery systems, and engineered nanoparticles, hold potential for enhancing the bioavailability and therapeutic efficacy of treatments.

## 1 Introduction

Glioblastoma (GB) is the most common and aggressive adult primary malignant brain tumor. First-line FDA-approved treatment relies on a multimodal approach of surgery, radiation, temozolomide (TMZ) chemotherapy, and tumor-treating fields. Unfortunately, the prognosis remains poor, with a median overall survival (mOS) of 20.9 months and a median progression-free survival (mPFS) of 6.7 months (1). The therapeutic outcomes are even less favorable in recurrent disease, where there is no standard of care, and the mOS from the first recurrence is around 6.5 months (2). Currently, there are only limited FDA-approved drugs for GB, such as TMZ, Bevacizumab, CCNU, and BiCNU, highlighting the urgent need for new treatment options for GB. One of the most promising approaches in cancer therapy is implementing next-generation sequencing (NGS) techniques to uncover actionable mutations that can be targeted in a tissue-agnostic fashion. In that regard, repurposing existing anticancer medications offers a potentially efficient and effective tool to discover new therapeutic agents for GB.

Here, we will discuss the previously explored repurposing efforts in the treatment of GB based on targeting two of the three most altered signal transduction pathways in GB: Receptor tyrosine kinase (RTK) and cyclin-dependent kinase (CDK)-retinoblastoma (Rb) (3). Approximately 57-60% of glioblastomas exhibit alterations in the RTK/PI3K pathway, with EGFR amplification or mutation being the most common, occurring in about 40-50% of cases (3). The CDK-Rb pathway is also frequently altered, with aberrations occurring in about 78-80% of cases, often involving the loss of CDKN2A, amplification of CDK4 or CDK6, and/or inactivation of the Rb1 protein. There are no validated targeted therapeutics for the murine double minute 2 (MDM2)-p53 pathway, the third commonly altered pathway in GB or other malignancies, primarily due to its crucial role in normal cell functions (4).

Despite an increased understanding of GB tumor biology with discoveries of potential targets, the unique challenges posed by this aggressive tumor continue to thwart treatment benefits. The tumor microenvironment (TME) of GB is highly heterogeneous and exceptionally dynamic, creating a landscape where cancer cells can evade therapies and rapidly adapt to treatment pressures. Compounding this complexity is the formidable blood-brain barrier (BBB), which acts as a gatekeeper, severely limiting the delivery of therapeutic agents to the tumor bed. Moreover, the inherent risk and challenges with accessing GB tissue at recurrence further hamper the ability to tailor treatments to individual patients, making it difficult to combat this relentless disease.

However, hope lies in innovative approaches designed to overcome these barriers. Cutting-edge techniques aimed at enhancing the detection of cancer biomarkers are on the horizon, offering the potential for more precise targeting of GB. Additionally, novel drug delivery vehicles, such as nanoparticles (NPs), are being developed to penetrate the BBB and deliver therapies directly to the tumor site. These advancements not only hold promise for improving treatment outcomes but also represent a bold step forward in the fight against GB.

## 2 RTK pathway inhibitors

### 2.1 Epidermal growth factor receptor-targeted agents

Anti-EGFR tyrosine kinase inhibitors (TKIs) have been a focal point in GB treatment trials due to the high rates of EGFR alterations, reaching ~60% in GB (5). Gefitinib was the first anti-EGFR TKI tested in GB. Various trials evaluated the role of gefitinib monotherapy in recurrent glioblastoma (rGB) treatment, showing good safety data but without promising efficacy (6, 7). The use of gefitinib with other RTK pathway inhibitors (8) and in newly diagnosed GB (nGB) treatment (9) was similarly inefficacious.

Erlotinib demonstrated limited efficacy in the treatment of GB. Early clinical trials indicated minimal activity, primarily because the drug couldn’t penetrate the blood-brain barrier (BBB) effectively and because the tumor growth wasn’t largely dependent on the targeted pathway (10). Subsequent trials exploring combinations of erlotinib with various chemotherapeutic agents, such as carboplatin, failed to yield significant improvements in PFS or OS in rGB (11). Additionally, while the addition of receptor tyrosine kinase (RTK) pathway inhibitor sirolimus, the mammalian target of rapamycin (mTOR) inhibitor temsirolimus, Ras/Raf/mitogen-activated protein kinase (MAPK) inhibitor sorafenib, and the anti-angiogenic agent bevacizumab was well tolerated, these combinations did not translate into clinically meaningful survival benefits (12–15). Furthermore, erlotinib demonstrated limited efficacy in treating newly diagnosed glioblastoma (nGB) (16).

Lapatinib, a dual inhibitor of EGFR and human epidermal growth factor receptor 2 (HER2), is the most frequently tested EGFR inhibitor in GB trials. However, its activity has been minimal when used as alone (17) and in combination with TMZ (18) or with pazopanib in rGB (19). In the nGB setting, pulsatile dosing of lapatinib in conjunction with TMZ chemoradiation was well-tolerated and showed promise in a phase 2 study, although further clinical data is currently lacking to establish its efficacy (20). The anti-EGFR monoclonal antibody cetuximab has not consistently outperformed existing GB therapies (21, 22). Nevertheless, a subgroup analysis from a phase 2 trial involving rGB patients with EGFR mutations revealed that tumors with EGFR amplification but without the EGFR variant III mutation experienced a statistically significant increase in progression-free survival (PFS) of 3.03 months compared to 1.63 months (p = 0.006), with a trend toward improved overall survival (OS) of 5.56 months versus 3.97 months (p = 0.12) (22). This suggests that certain EGFR mutations may confer a selective advantage in anti-EGFR treatment for GB.

Nimotuzumab is another anti-EGFR monoclonal antibody that has shown some promise, particularly when combined with radiation or TMZ chemoradiation. A phase 2 study reported an improved median overall survival (mOS) when nimotuzumab was combined with radiation in patients with high-grade glioma, showing a mOS of 17.76 months compared to 12.63 months in the placebo plus radiation group (23). Another phase 2 trial yielded similar positive outcomes with nimotuzumab (24). However, a subsequent phase 3 trial that combined nimotuzumab with TMZ chemoradiation did not replicate these survival benefits (25), suggesting a nuanced potential for monoclonal antibodies in targeted GB therapy, dependent on specific patient genetic profiles.

Recent preclinical studies utilizing *in vitro* glioblastoma stem cells (GCS) models and GB orthotopic xenograft model with EGFR variant III showed antitumor activity along with inhibition of EGFR downstream signaling pathway for the third-generation EGFR inhibitor osimertinib (26). Initial clinical studies of osimertinib in GB treatment are promising. Two small retrospective studies on rGB patients with EGFR mutations showed some benefit when used as monotherapy (27) and in combination with bevacizumab (28). These promising findings indicate that osimertinib’s primary advantage over earlier generations of EGFR inhibitors in GB therapy lies in its superior ability to penetrate BBB.

### 2.2 Platelet-derived growth factor receptor-targeted agents

Imatinib, a drug primarily approved for leukemia, has been explored for its potential in treating rGB with mixed outcomes. An initial phase 2 study combining imatinib with hydroxyurea demonstrated some antitumor activity, with a median PFS of 14.4 weeks and 9% of patients achieving radiographic responses (29). However, subsequent trials found no significant clinical benefit with imatinib, either as a monotherapy or in combination with hydroxyurea (30–32). Similarly, other early-phase trials that combined imatinib with TMZ (33) or the vascular endothelial growth factor receptor (VEGFR) inhibitor vatalanib (34) reported minimal clinical activity. While neoadjuvant administration of imatinib resulted in detectable drug levels in brain tissue, it had a limited impact on tumor proliferation and patient survival (35, 36), highlighting the drug’s limited efficacy in this context.

Dasatinib, another leukemia-approved drug, was tested in rGB with minimal success. A retrospective study combining dasatinib with bevacizumab showed little activity (37), and further trials involving dasatinib with CCNU highlighted significant hematologic toxicities (38), curtailing additional studies with this combination. More focused clinical trials on rGB harboring activation or overexpression mutations of dasatinib targets, such as SRC, c-kit, EPHA, and PDGFR, also indicated insufficient clinical benefits, even with pulse-dosing strategies combined with bevacizumab (39).

Ripretinib (DCC-2618), an innovative type 2 tyrosine switch control inhibitor of the KIT and PDGFRA activating mutations, showed some potential in an early-phase study in which five high-grade glioma patients were enrolled. One of the two GB patients carrying triple amplification of PDGFRA, KIT, and KDR (4q12 amplicon) showed a remarkable 94% tumor reduction and survived through over 20 cycles. However, larger-scale evidence is still lacking (40).

Despite the theoretical promise based on their successful oncological applications elsewhere, none of those above PDGFR-targeted agents demonstrated substantial benefits in GB, particularly in recurrent settings.

### 2.3 VEGFR-targeted agents

Cabozantinib, a multikinase inhibitor targeting targets Met, VEGFR, and Axl, has been approved for different cancers. It has been tested in rGBs with mixed results. In large phase 2 trials, cabzantinib demonstrated reasonable tolerance and some clinical activity in rGB patients who were naïve to antiangiogenic therapy (41) or those previously exposed to an antiangiogenic agent (42).

Another multi-kinase inhibitor, Sunitinib, showed limited antitumor activity across several GB trials. Its use with irinotecan resulted in moderate toxicities and insufficient clinical effectiveness (43, 44), leading to the early termination of a subsequent phase 2 trial (45). Another phase 2 trial using sunitinib monotherapy in non-resectable nGB also failed to show antitumor activity (46). Despite these setbacks, interest in sunitinib continues with ongoing trials exploring different dosing strategies in the rGB setting.

Pazopanib, approved for sarcoma, is also a multikinase inhibitor targeting VEGFR and PDGFR. It has similarly struggled to demonstrate efficacy in GB. An initial trial using pazopanib monotherapy in rGB failed to prolong PFS, and a subsequent trial combining pazopanib with lapatinib in rGB yielded questionable antitumor activity with 0% and 15% PFS rates in PTEN/EGFRvIII-positive and PTEN/EGFRvIII- negative cohorts respectively (19). A complex combination therapy trial combining pazopanib and four other drugs showed promising clinical response rates: complete response (CR) in 18.2.%, partial response (PR) in 36.3%, and stable disease (SD) in 27.3% of patients. However, issues with patient compliance halted further exploration of this regimen (47). Additional studies pairing pazopanib with topotecan and bevacizumab also did not meet the anticipated outcomes, recording poor mPFS and mOS rates compared to historical controls, as reported in preliminary results on clinicaltrials.gov (48).

### 2.4 Fibroblast growth factor receptor-targeted agents

The frequency of FGFR mutations in GB is relatively low, resulting in limited use of FGFR inhibitors in this disease. However, various preclinical studies showed that FGFR signaling has a significant impact on GB progression (49).

Infigratinib monotherapy was tested on twenty-six rGB patients in a phase 2 trial, and it showed limited efficacy overall. However, durable disease control was observed in subgroups of patients harboring FGFR1 or FGFR3 point mutations or with FGFR3-TACC fusion mutation (50).

Two separate FGFR-mutated solid malignancy basket trials with erdafitnib (51) and pemigitinib (52), including thirty-two and twelve glioma patients, respectively, showed promising clinical benefits that have not been verified in subsequent trials yet.

### 2.5 PIK/Akt/mTOR pathway inhibitors

The PI3K/Akt/mTOR pathway, mutated in approximately 45.6% of GB cases, has been one of the key pathways implicated in tumorigenesis.

Paxslisib is a selective small-molecule PI3K inhibitor used in two glioma trials. A phase 1 study on forty-seven recurrent high-grade glioma patients showed reasonable safety and promising efficacy, with 40% having SD (53). A subsequent multi-center phase 2 study on thirty patients with O^6^-methylguanine-DNA methyltransferase (MGMT) promoter unmethylated nGB showed encouraging survival data with median PFS and OS of 8.6 months and 15.7 months, respectively (54).

Buparlisib, a pan-PI3K inhibitor, has been the focus of several clinical trials in GB. Although it showed good brain penetrance and tolerability in a phase 2 trial for rGB with PI3K pathway activation mutations, its efficacy was limited, with only a small fraction of patients achieving PFS at six months (55). Subsequent trials combining buparlisib with other therapies like bevacizumab, carboplatin (56), or the c-met inhibitor capmatinib (57) did not demonstrate superior efficacy over monotherapy or existing treatments.

Another therapeutic approach involved the mTOR inhibitors temsirolimus and everolimus, both of which have been extensively evaluated in GB in various clinical settings. An early phase trial using temsirolimus in rGB showed promising results, with 36% of participants showing radiographic responses and significantly longer time to progression than non-responders (58). However, the drug failed to meet efficacy endpoints in subsequent studies, including combinations with TMZ chemoradiation (59) and sorafenib (60). These combinations often resulted in increased toxicity, most notably severe hematologic toxicity, and increased infection risk (61).

Everolimus use with TMZ and radiation in nGB has shown reasonable tolerability in phase 1 trials (62–64). However, subsequent phase 2 trials did not demonstrate a significant survival benefit over historical controls (65) or over TMZ arm in a randomized trial (66). A phase 2 study for nGB treatment with concurrent TMZ, bevacizumab and radiation therapy followed by adjuvant treatment with bevacizumab/everolimus showed a favorable response with median PFS of 11.3 months but not mOS benefit compared to historical control. Additionally, the radiographic objective response rate (ORR) of 61% could have been influenced by the use of bevacizumab, considering its known radiographic effects (67).

### 2.6 Pan-kinase inhibitors

Anlotinib is a multi-kinase inhibitor targeting VEGFR, PDGFR, and FGFR. In a phase 2 study involving 21 patients with recurrent rGB, anlotinib combined with temozolomide demonstrated efficacy, achieving a median progression-free survival (PFS) of 7.3 months and a median overall survival (OS) of 16.9 months (68). Additionally, anlotinib showed promising activity in patients with MGMT promoter-unmethylated nGB when used in place of temozolomide in a phase 2 study of 32 patients (69).

Regorafinib, a pankinase inhibitor, was initially tested as monotherapy on rGB patients in a phase 2 trial, which showed a survival benefit when compared to CCNU (70). Recently, a large multi-center prospective observational trial on 190 rGB patients showed similar promising mOS and better drug tolerability compared to that seen by Lombardi et al. (71).

## 3 CDK-Rb targeting agents

Targeting this pathway in oncology has been primarily limited to CDK4/6 inhibitors.

Abemaciclib activity in rGB was assessed in a basket trial involving seventeen GB patients and showed limited effectiveness. A subsequent phase 2 trial on thirty-two rGB with documented CDK mutations showed SD in 35.5% and PR in 3.2% of the patients (72). More recently, abemaciclib was tested on seventy-three nGB patients in a phase 2 study, resulting in improved PFS compared to standard of care (HR 0.72; one-sided p =0.046), suggesting some potential for this drug in specific GB populations, but it failed to demonstrate significant overall survival (OS) benefit (73).

Palbociclib role in GB has been less encouraging. A phase 2 trial on heavily pretreated rGB patients noted adequate pharmacokinetics but ultimately showed limited efficacy, leading to the trial’s termination (74). No further studies are currently investigating its role in GB.

Ribociclib, another CDK4/6 inhibitor, also displayed minimal clinical activity in a phase 0/2 surgical trial on rGB patients (75). The trial identified upregulation of the mTOR pathway as a potential resistance mechanism, suggesting the addition of mTOR inhibitor as a potential strategy to enhance ribociclib effectiveness in GB treatment.

## 4 Repurposing targeted drugs in GB: challenges and solutions

Below described are the main barriers to GB targeted therapy, which is a field largely dependent on repurposed drugs, with a few exceptions outside the scope of this review.

### 4.1 Complexity of GB biology

Intra-tumoral heterogeneity, which has also been previously described in GB using single-cell RNA-seq profiling (76), complicates accurate targeted therapy in several ways. Different regions within the tumor exhibit distinct genetic, epigenetic, and transcriptional profiles. As a result, tissue samples, particularly those obtained through biopsy, may not capture the full spectrum of mutations within the tumor, leading to suboptimal treatment strategies that fail to target all tumor cell populations. Furthermore, this variability complicates the identification of reliable therapeutic targets, as a target found in one tumor region may be absent in another, increasing the likelihood of treatment failure. Resistance mechanisms also play a critical role, with certain cell populations potentially being resistant to specific therapies due to their unique genetic or epigenetic characteristics. This resistance can lead to disease progression and recurrence, especially as therapy may select for these resistant clones over time. While single-cell RNA-seq provides valuable insights into tumor heterogeneity, its application in clinical practice is limited by the complexity and resources required for its clinical use.

**Figure 1:**
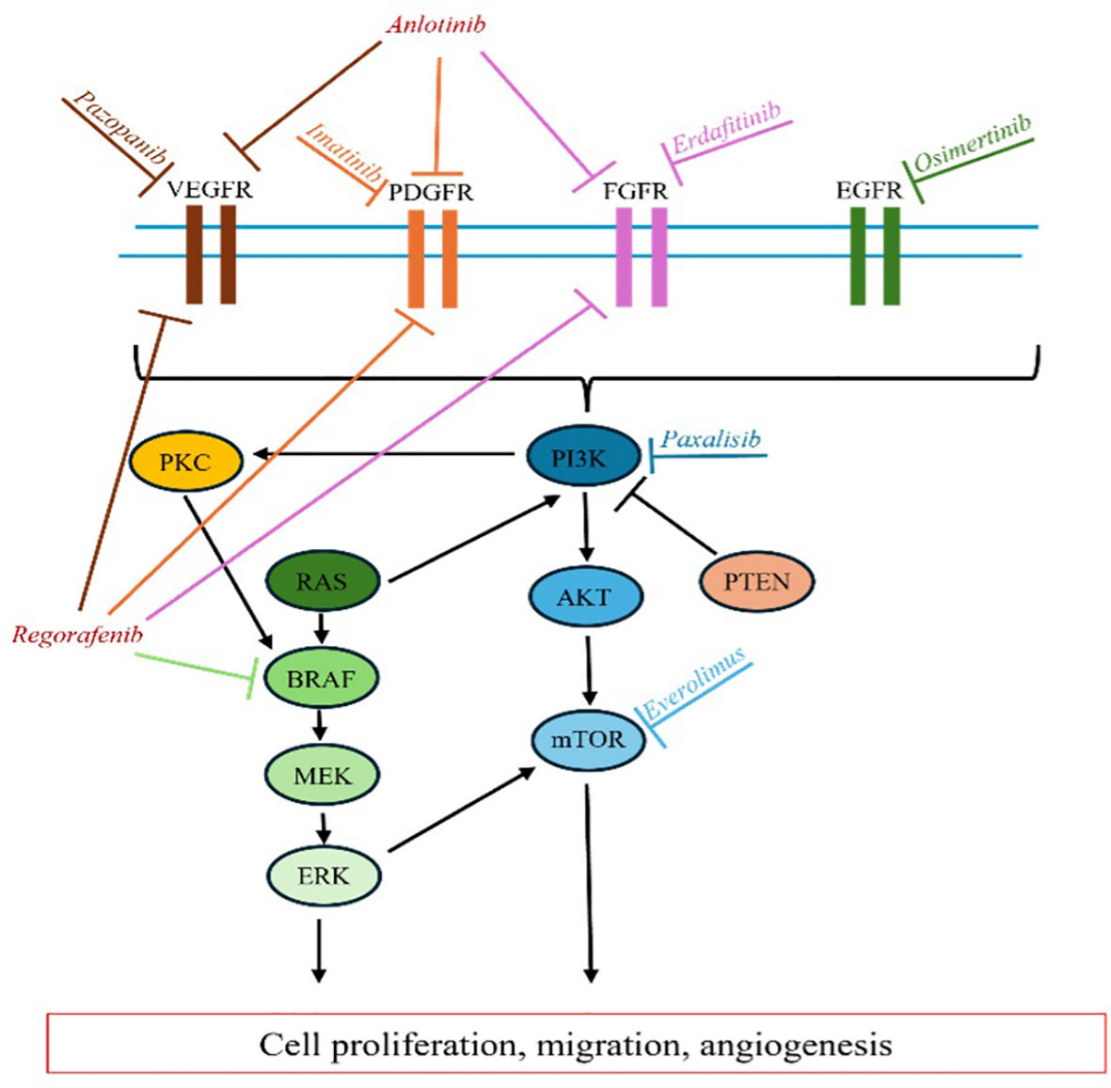
RTK Pathway. Overview of targeted inhibition of various RTK signaling enzymes.

Redundant signaling pathways in gliomagenesis limit the effectiveness of targeted therapies that focus on a single gene or pathway, even when the tumor’s complete molecular profile is known. Combination strategies that target multiple actionable mutations within a tumor could potentially enhance the efficacy of targeted therapies for GB. However, the potential benefits of such approaches must be carefully weighed against the risk of cumulative toxicities associated with combination treatments. Additionally, our understanding of the role of downstream mutations within gliomagenesis signaling pathways remains incomplete. For example, PTEN mutations, which occur downstream of EGFR signaling, have been identified as a resistance factor in anti-EGFR therapy for GB, highlighting the complexity of these pathways and the challenges in developing effective targeted treatments (77). **Figure 1** further illustrates the intricate and overlapping signaling cascades within the RTK pathway, emphasizing the complexity involved in the context of the various targeted therapeutics discussed above.

Another challenge is the lack of a consensus on what level of increased expression is considered significant enough to influence clinical decisions. NGS platforms routinely provide the percentage of overexpression of an amplified gene, but there are no studies that have stratified clinical response to a drug by the fold-increase of its target. Retrospective analysis of clinical trial data using targeted therapeutics based on survival by target fold-increase may help refine personalized therapies.

Reliance on a single molecular profiling platform, such as NGS, can diminish the importance of other platforms in identifying personalized treatment response signatures. For instance, methylation profiling of GB specimens is currently the only sequencing method that can identify a subset of IDH-wild type gliomas. This subset represents a negative prognostic marker that is sufficient to diagnose GB in a glioma, regardless of its histopathological grade (78). On the other hand, methylation of MGMT gene promoter is associated with a more favorable prognosis (79). High-throughput drug screening combined with pan-omic molecular profiling of response can help generate relevant predictive biomarker libraries (80), resulting in a more nuanced approach to targeted therapy, one that is not single actionable mutation-based. Such efforts can also help inform future clinical trial design.

### 4.2 Dynamic evolution of the tumor

GB is a dynamic tumor that continuously evolves in response to therapeutic interventions like radiation and chemotherapy. This evolution is driven by clonal diversity within the tumor, where different cell populations harbor distinct genetic mutations. As treatments impose selective pressures, sensitive clones are eliminated, while resistant clones survive and proliferate. For example, radiation and chemotherapy, such as alkylating agents, work by imposing lethal DNA damage. However, clones with efficient DNA repair mechanisms can selectively survive through radiochemical stress and flourish in less competitive environments. On the other hand, the increased mutation burden from radiation or chemotherapy can potentially lead to the emergence of new mutations that confer resistance to the therapy (81). This dynamic nature means that the tumor’s mutational profile at recurrence differs from its profile at diagnosis, and the problem is exacerbated by limited access to GB tissue at recurrence, as re-resection is not always safe or preferred. The failure of most targeted therapy trials for rGB can be attributed to their focus on the tumor’s initial mutational profile, which may not reflect the tumor’s current genetic state due to its evolution over time. Advances in liquid biopsy techniques for non-invasive profiling of GB can help to overcome this challenge and will be discussed in more detail in the next section.

For nGB, the presence of FDA-approved standard chemotherapy (TMZ) limits the application of new therapies and delays the approval of new drugs for nGB. Advances in clinical trial design for these patients fall under one of three categories:

1. Combining the experimental drug with TMZ so as not to deprive patients of standard of care treatment.
2. Using an experimental drug instead of TMZ in the setting of unmethylated MGMT promoter which results in high expression of the DNA-repair enzyme MGMT. Overexpression of MGMT results in decreased response to alkylating agents such as TMZ, as evidenced by the significantly improved survival outcomes in the MGMT promotor methylated group (Median PFS 19 mo *vs*. 6 months; p=.014) when adjuvant TMZ, lomustine, and radiation therapy was used (82).
3. Utilizing adaptive trial platforms to accelerate drug discovery in an efficient and cost-effective manner. This approach involves continuously adjusting multiple treatment arms, including adding new ones and terminating others early based on emerging data, all within a single master protocol. Response-adaptive randomization allows trials to progress through different phases more quickly and facilitates the rapid identification of promising therapies and response biomarkers. Currently, two major adaptive trials in GB, AGILE and INSIGhT, have successfully increased enrollment rates by leveraging this methodology (83)

Lastly, the frequency of an actionable mutation within a tumor, as indicated by variant allele frequency (VAF) in NGS reports, varies between different GB specimens. However, targeted therapy trials almost never include a VAF cutoff as an eligibility criterion. Consequently, these trials’ perceived low response rate may be diluted by a subset of patients with low VAF, highlighting the need for detailed subgroup analysis upon trial completion.

### 4.3 GB microenvironment

#### 4.3.1 BBB

BBB is a critical and complex structure that serves as a dynamic interface between the bloodstream and the CNS. It is primarily composed of endothelial cells, basement membrane, pericyte, and astrocyte. The endothelial cells are tightly connected by intracellular junctions that play a crucial role in maintaining the highly selective permeability of the BBB, allowing it to regulate the movement of ions, molecules, and cells between the blood and the neural tissue (84, 85). This selective permeability is essential for maintaining CNS homeostasis, protecting the brain from toxins, pathogens, and immune cells, and regulating the influx and efflux of nutrients and other compounds necessary for brain function. The BBB’s integrity is hallmarked by its high transendothelial electrical resistance (TEER), which restricts the passage of most water-soluble compounds, including polar drugs (86). Only small, lipophilic molecules, such as oxygen and carbon dioxide, can passively diffuse across the BBB. Additionally, specific transporters and receptors on the endothelial cells facilitate the selective transport of essential molecules like glucose, amino acids, and nucleosides, while actively effluxing potentially harmful substances, including many therapeutic drugs, back into the bloodstream.

This highly selective and regulated nature of the BBB presents a significant challenge in the treatment of GB. The barrier limits the delivery of therapeutic agents, particularly large or hydrophilic molecules, into the brain. Most anti-neoplastic drugs, which are often hydrophilic and large, cannot cross the BBB efficiently due to their size and polarity (87, 88). Furthermore, the presence of active efflux transporters like P-glycoproteins exacerbates this challenge by pumping out drugs that manage to penetrate the endothelial cells (89). Moreover, the BBB’s integrity is not uniform throughout the GB tumor (90). While the BBB may be compromised in some regions of the tumor, allowing partial drug penetration, other areas may still have an intact barrier, further complicating effective drug delivery. This heterogeneity in BBB disruption within GB tumors means that even if a therapeutic agent reaches some parts of the tumor, it may not reach all areas, leading to incomplete treatment and potential recurrence. Consequently, innovative strategies are required to either bypass or transiently disrupt the BBB to improve drug delivery and therapeutic efficacy in GB treatment. One strategy involves incorporating a window-of-opportunity component into surgical clinical trials. In this approach, patients receive the experimental drug before the tumor resection. Pharmacodynamic studies on the resected tumor can then determine whether the drug crossed the blood-brain barrier (BBB) and performed its expected function. This helps differentiate between a mechanistic failure and a delivery failure of the experimental drug (91). BBB disruption is another strategy that will be discussed in more detail below.

#### 4.3.2 Hypoxia

GB TME features a necrotic core primarily formed due to high cell density or vaso-occlusive events leading to hypoxia, a pervasive feature in GB. The hypoxic niches contribute to the development of therapy resistance to conventional chemotherapy, which often relies on oxygen to generate reactive oxygen species (ROS) that damage cancer cells. This results in increased expression of hypoxia-inducible factors (HIFs), which promote angiogenesis (via VEGF upregulation) and invasion (92, 93). In the context of radiotherapy, hypoxic tumor cells exhibit increased resistance due to their impaired ability to produce damaging oxygen radicals upon irradiation (94). Additionally, HIFs help mitigate the DNA damage caused by radiotherapy, further reducing its effectiveness (95).

#### 4.3.3 Acidosis

Tumor acidosis crucially impacts the effectiveness of various therapeutic interventions by modulating the TME and promoting oncogenesis. It enhances the expression of glioma stem cell (GSC) markers, fostering tumor growth through paracrine actions that involve angiogenic factors controlled by HIFs, particularly HIF-2α (96). Acidosis also heightens autophagic activity linked to the maintenance and aggressiveness of GSCs (97). Additionally, it supports tumor invasion by activating cathepsin L, which converts plasminogen into plasmin, leading to the degradation of key extracellular matrix proteins and activation of latent matrix metalloproteinases (98). It also compromises the efficacy of chemotherapeutics, particularly weak base drugs like doxorubicin and vincristine, through ion trapping that reduces their intracellular concentration and by increasing the efflux activity of the p-glycoprotein (99–101). Moreover, acidosis facilitates tumor immune escape and resistance to immunotherapy by impairing CD8+ T lymphocytes, reducing their cytokine secretion and expression of critical receptors, thus dampening key immune signaling pathways (102, 103). It also lowers the production of vital immune effectors by T cells and monocytes, enhances the number of myeloid-derived suppressor cells (MDSCs), and inhibits the cytotoxic functions of NK and NKT cells (104).

#### 4.3.4 Glutathione

Elevated glutathione levels in GB lead to reduced oxidative stress which is crucial for disease progression (105). Although increased glutathione protects healthy cells from oxidative stress, it concurrently promotes resistance to many chemotherapeutics which exert their cancer killing properties by ROS production. This is further highlighted by studies showing that resistant cells had higher levels of glutathione and lower levels of ROS than TMZ-sensitive cells (106, 107).

#### 4.3.5 Altered drug mechanism of action

Repurposed drugs may exert their effect on the CNS tumor differently from their actions in the originally FDA-approved tumor type (108, 109). Many drugs repurposed for GB treatment, like cabozantinib, inhibit multiple pathways. While they are primarily known as VEGF inhibitors, their role as multi-targeted tyrosine kinase inhibitors can lead to a range of effects, resulting in a broader range of biological effects. This variability in drug action within the unique CNS tumor microenvironment further complicates the therapeutic efficacy and predictability of these agents (110).

The cumulative effect of all these features is a selective pressure favoring the growth of resistant clones, complicating future therapies, whether targeted or otherwise.

## 5 Emerging diagnostic tools: liquid biopsy

As previously mentioned, the failure of drug repurposing efforts in GB can be attributed to several factors. Tumor heterogeneity, characterized by the presence of multiple clones, increases the likelihood of drug-resistant clones, resulting in treatment failure or recurrence. Even if the tumor initially responds to treatment, tumor cells may evolve into different phenotypes with new mechanisms of therapeutic resistance. In clinical practice, disease monitoring predominantly relies on imaging modalities, limiting the ability to observe real-time tumor dynamics and variations in biomarkers. While tumor biopsy at recurrence is an option, it is infrequently performed due to its morbidity risks and the lack of clear benefit. It remains uncertain whether molecular characterization of recurrent tumors provides a definitive survival advantage in patients treated with targeted therapies. In that regard, liquid biopsy rapidly emerges as a promising non-invasive diagnostic tool, showing encouraging results.

By analyzing molecular biomarkers in bodily fluids, liquid biopsies provide a real-time snapshot of the tumor’s genetic landscape without the need for invasive surgical procedures. This approach can detect changes in the tumor’s mutational profile over time, allowing for more precise and adaptive treatment strategies that respond to the tumor’s evolving nature. Liquid biopsies offer a promising tool to enhance the effectiveness of targeted therapies and improve the prognosis for GB patients by enabling continuous monitoring and timely adjustments to therapeutic interventions.

**Figure 2:**
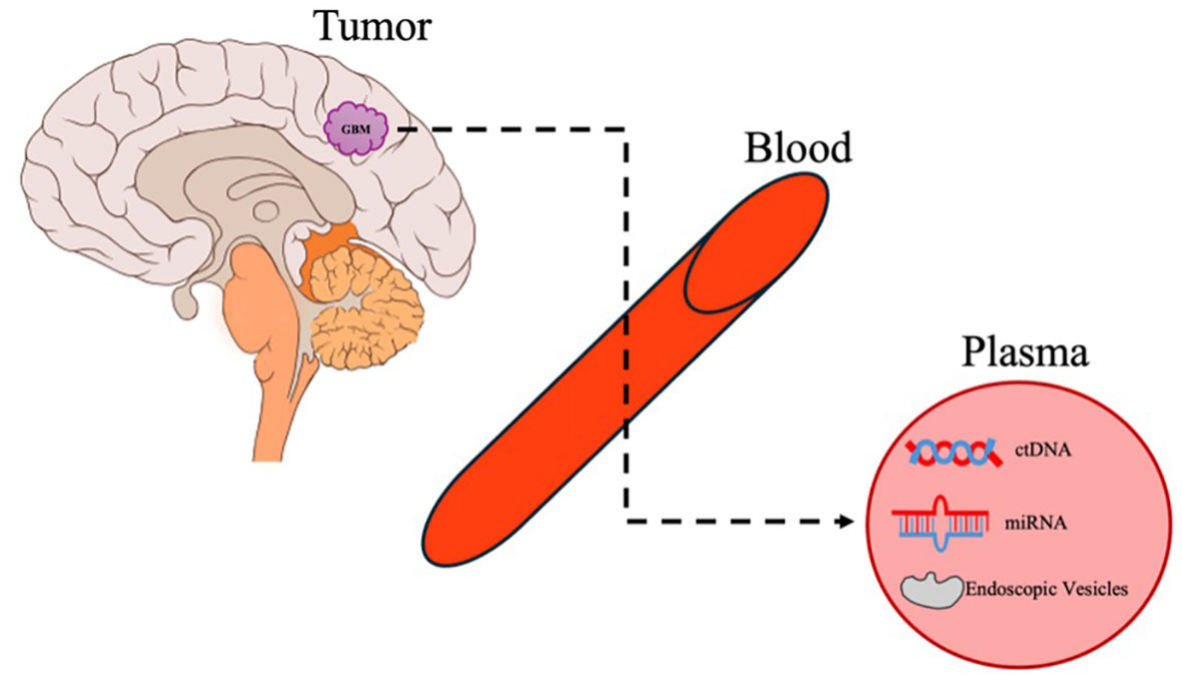
Liquid biopsy. An overview of the release of GB molecular biomarkers and cargo into the systemic circulation and its detection in plasma.

While the field of liquid biopsy in GB began with studies on circulating tumor cells, there are no validated or reproducible studies on isolating them. Therefore, we will focus here on the three main molecular markers studied in liquid biopsy (**Figure 2**).

### 5.1 ctDNA

The detection of circulating tumor DNA (ctDNA) in glioma patients varies significantly between blood and cerebrospinal fluid (CSF). The BBB limits ctDNA detection in blood to less than 10% of glioma patients, primarily due to ctDNA’s inability to effectively penetrate the BBB (111). In contrast, ctDNA levels in CSF are generally higher (112, 113), as it is directly shed into the CSF from the tumor. However, monitoring treatment response by serial CSF ctDNA measurements requires repeated lumbar punctures and exposes patients to potentially unjustified morbidities.

Peripheral blood is a less invasive option for serial monitoring, facilitating longitudinal monitoring studies, but it presents several challenges. The rapid clearance of ctDNA with a half-life of about 1.5h (114) and the small size of ctDNA fragments, which may not include relevant genetic alterations, contribute to plasma ctDNA’s low sensitivity. However, recent advances in NGS have increased the sensitivity of their detection. According to Piccioni et al., the NGS panel could detect ctDNA mutation in blood from 50% of all brain tumor patients and 55% of GB patients (115), allowing for measurement of GB’s mutational profile evolution during treatment. A more recent pilot study enrolling ten glioma patients utilizing the CAPP-seq-based NGS technique reported a detection rate of up to 93.8% plasma samples with successful tracking of change in mutation profiles (116). The serial ctDNA analyses detected an emergence of mismatch repair gene *MSH2* and *MSH6* gene mutations, which is associated with hypermutation and potential development of TMZ resistance during treatment with TMZ. Another study utilizing a droplet digital PCR technique reliably detected the IDH1 mutation with 84% sensitivity in cross-comparison with tissue mutations (117). TERTp C228T mutation was detected in 88% of patients, and EGFRvIII mutation was detected in 71% of patients.

### 5.2 miRNA

There have been several studies exploring the role of circulating miRNA in GB. Differentially expressed miRNAs were identified in several studies comparing the plasma of GB patients to that of healthy controls (118–120). Circulating miRNAs can also serve as biomarkers correlating with OS and PFS in GB patients (121, 122). However, the clinical implications of these findings in relation to treatment strategies warrant further exploration.

### 5.3 Extracellular vesicles

Extracellular vesicles (EVs) are small membrane-bound vesicles released by cells under both normal and abnormal conditions, playing a crucial role in cell-to-cell communication. These vesicles transport genetic materials like DNA, mRNA, and miRNA across the body, influencing the behavior and phenotype of distant cells, including endothelial cells (123, 124).

EVs originating from gliomas or other cells in the tumor microenvironment appear to play crucial roles in tumor cell proliferation, invasion, malignancy, and drug resistance (125). Cancer cells release a greater number of EVs with differing protein and RNA contents, compared to non-malignant cells (126). These EVs facilitate communication with surrounding cells to alter the TME by influencing the behavior of local and recruited stromal cells, contributing to the creation of a tumor-supportive environment that enhances angiogenesis, immunosuppression, and the malignant transformation of cancer cells (127). Tumor-derived EVs can also enter into the circulation and prepare distant organs for metastasis by creating favorable conditions for tumor cell growth (128). This process, known as pre-metastatic niche formation, involves steps such as inducing vascular leakiness, altering stromal components, and suppressing the immune system. These interactions underscore the significance of EVs in the progression and maintenance of cancer.

From a diagnostic standpoint, EVs are increasingly recognized for their potential in identifying tumor molecular signatures, particularly due to their ability to traverse an intact BBB (129). Manda et al. reported that variant EGFR RNA transcripts were detected with similar frequency in GB tissue (39.5%) and their matched serum exosomes (44.7%). The presence of circulating exosomal EGFRvIII variants correlated with poor outcomes (130). Studies on miRNA contents of EVs revealed that the exosomal levels and types of miRNA within the EVs were associated with the aggressive potentials of the GB (131). For example, an *in vitro* functional study using glioma cell lines has indicated that miR-221 silencing can reduce cell proliferation, migratory potential, and resistance to temozolomide (132). In addition, exosomal levels of miR-221 were increased in parallel with glioma grades. Another study reported that syndecan-1 plasma EVs could distinguish between low-grade and high-grade gliomas with a sensitivity and specificity greater than 70% (133).

However, challenges remain. We still lack an understanding of the mechanisms that drive the RNA incorporation into EVs of various sizes and types in different RNA concentrations. The technical challenges of isolating purified tumor-specific EVs of different sizes in high-yield continue to complicate the interpretation and utility of these biomarkers in clinical settings.

Liquid biopsy offers significant potential in enhancing the management of GB by providing a non-invasive method to monitor treatment response in real-time, tracking changes in tumor-derived biomarkers. By identifying specific genetic mutations and alterations, liquid biopsies can guide targeted therapies, facilitating more personalized and effective treatment strategies. However, the clinical implementation of liquid biopsy in GB is still in its early stages. There is a need for standardized methodologies across laboratories to ensure consistent and reliable results. Larger prospective studies are required to validate the clinical utility of liquid biopsy biomarkers in GB.

## 6 Strategies to improve drug delivery to GB

Effective drug delivery to the CNS is significantly hindered by the BBB, posing a substantial challenge in GB treatment. The BBB is a multi-layered cellular physical barrier composed of endothelial cells, astrocytes, and pericytes, which effectively prevent the diffusion of small molecules. It facilitates the exchange of essential nutrients and metabolites through a selective transport system. Even if small molecules manage to penetrate the BBB, they are often actively transported back out via efflux pumps (134). Overcoming this barrier to enhance bioavailability is a critical area of ongoing research.

### 6.1 Focused ultra-sound

FUS has been investigated as a method to transiently and non-invasively increase the permeability of the BBB, thereby enhancing the delivery of therapeutic agents to GB tissue. This technique involves the use of pulsed ultrasound waves in conjunction with microbubbles, which oscillate and cause transient disruption of the BBB. Preclinical studies have demonstrated that FUS can improve the concentration of chemotherapeutic agents such as TMZ in brain tissue, leading to prolonged survival in animal models (135, 136). Adding MRI to FUS (MR-guided FUS) further improves the precision of the drug delivery, thus minimizing damage to healthy tissue. Clinical trials have begun to explore the safety and efficacy of FUS in humans, with early results indicating that FUS can be safely performed and may improve drug delivery and patient survival (137, 138).

Animal studies have shown that FUS can improve the penetration of immunotherapy into brain tumors, the immune response against the cancer cells, and survival outcomes (139, 140). The clinical use of immunotherapy in GB treatment is currently not validated with debatable early study outcomes.

FUS is actively investigated for its use in direct tumor ablation by delivering high-energy ultrasound waves. Initial clinical studies using MR-guided FUS demonstrated precise ablation of brain tumors after craniotomy (141). However, significant attenuation of ultrasound waves by the skull, significant damage to healthy brain tissue, and lack of clinical validity currently limit its use.

### 6.2 Implantable drug-delivery systems

The development of new extended-release drug-delivery vehicles led to several promising strategies for improving patient outcomes after tumor resection.

The pioneering work of Langer’s group in the 1980s led to the development of localized controlled-release therapies for GB, culminating in the FDA approval of the first implantable intra-cavity wafer, Gliadel, in 1996 (142). Gliadel, a polyanhydride-based wafer containing carmustine (BCNU), is designed for optimal drug release, achieving substantial polymer degradation within three weeks of implantation. Clinical trials demonstrated survival benefits for patients receiving Gliadel compared to placebo (13.8 months *vs* 11.6 months; p=0.018) (25). On the other hand, implantation is associated with several negative side effects, including seizures, vasogenic edema, meningitis, and impaired wound healing (143). It can also dislodge and cause micro-tears (144). Also, its content (BCNU) has a low diffusion rate (145). Therefore, it is no longer commonly used in clinical practice.

Following Gliadel’s approval, there has been a surge in the development of locally administered chemotherapeutic devices. Sheleg et al. explored a biodegradable polymer device loaded with cisplatin (146). Twenty 1.5 x 1.5cm polymer plates loaded with cisplatin with a drug density of 1mg/cm2 were implanted in the surgical bed after subtotal removal of GB. This strategy resulted in extended OS when administered with radiation therapy compared to radiation alone (427.5 *vs* 211.0 days; p = 0.00001) (146). Similarly, Di Mascolo et al. developed microfabricated PLGA meshes loaded with diclofenac and docetaxel, which effectively prevented tumor recurrence and significantly extended survival in orthotopic brain tumor mouse models, emphasizing the advantage of the meshes’ flexibility over solid films (147).

Technological advancement led to different designs of implantable drug-delivery systems, including hydrogel and microparticles. Hydrogel is a hydrophilic polymer network with high water contents that can be loaded with water-soluble biomacromolecules such as small molecules and NPs. For example, OncoGel, a type of thermoresponsive PLGA-PEG matrix hydrogel loaded with paclitaxel, transitions to a semisolid state at the body temperature once applied to the tumor bed and maintains drug release over six weeks (148). PLGA microparticles have been studied extensively, offering controlled release of anticancer drugs like 5-fluorouracil (149), which has shown to work synergistically with radiotherapy in enhancing survival in animal models (150).

There are no implantable targeted drug-delivery systems, likely due to cost considerations. However, as this technology advances and shows more promising data, it can potentially be applied to targeted therapeutics.

In the context of targeted therapy, only EGFR-targeting nanoparticles have been investigated in human studies. The first was a phase 1/2 trial involving fourteen patients with rGB. In this study, doxorubicin-loaded nanoparticles targeting EGFR were used to deliver the chemotherapeutic agent into GB tumor cells (155). While the maximum tolerated dose (MTD) was determined, the trial was prematurely terminated by the sponsor, leaving efficacy data unavailable. Similarly, a phase 1 study utilizing anti-EGFR doxorubicin-loaded immunoliposomes was conducted in nine rGB patients with EGFR amplification (155). This study confirmed the successful delivery of nanoliposomes to GB tissue; however, the small sample size and absence of a control group limited the assessment of therapeutic efficacy.

### 6.3 Nanoparticles

NPs can be engineered specifically to cross the BBB and target tumor cells directly, minimizing side effects. NPs, with their tunable physicochemical properties, can be loaded with various therapeutic agents to deliver the intended targets. The NPs can be tailored to have specific intrinsic (electronic, optical, and magnetic) and extrinsic (size, surface-to-volume ratio, or surface energy) characteristics to increase delivery efficiency, decrease off-target effects, and improve drug kinetics. These NPs act as “Trojan horses,” facilitating the delivery of drugs like doxorubicin and paclitaxel and biological molecules such as antibodies, DNA, and peptides directly to GB cells.

The surface of NPs can be modified with specific ligands that recognize and bind to receptors on endothelial cells lining the brain, facilitating their entry into the brain through mechanisms like receptor-mediated transcytosis. For example, the transferrin receptor (TfR) and low-density lipoprotein receptor (LRP1) are common targets on brain endothelial cells that NPs exploit to achieve transcytosis (151, 152). These receptors allow NPs to bypass the typical barriers posed by the BBB, improving the delivery efficiency of chemotherapeutics into the brain. Since the brain uptakes glucose via like the glucose transporter-1 (GLUT1), Anraku et al. utilized glycosylated micelles to transport bioactive substances via the GLUT1 transporter. The precisely calculated glucose density on the surface of the NP allowed the regulation of its distribution within the brain, thus successfully increasing the number of nanocarriers within the brain (153). Further, PEGylation of these NPs further prolongs their circulation time in the bloodstream, reducing protein interactions and enhancing their therapeutic efficacy (154).

In the context of targeted therapy, only EGFR-targeting nanoparticles have been investigated in human studies. The first was a phase 1/2 trial involving fourteen patients with rGB. In this study, doxorubicin-loaded nanoparticles targeting EGFR were used to deliver the chemotherapeutic agent into GB tumor cells (155). While the maximum tolerated dose (MTD) was determined, the trial was prematurely terminated by the sponsor, leaving efficacy data unavailable. Similarly, a phase 1 study utilizing anti-EGFR doxorubicin-loaded immunoliposomes was conducted in nine rGB patients with EGFR amplification (156). This study confirmed the successful delivery of nanoliposomes to GB tissue; however, the small sample size and absence of a control group limited the assessment of therapeutic efficacy.

## 7 Conclusions/future directions

**Figure 3:**
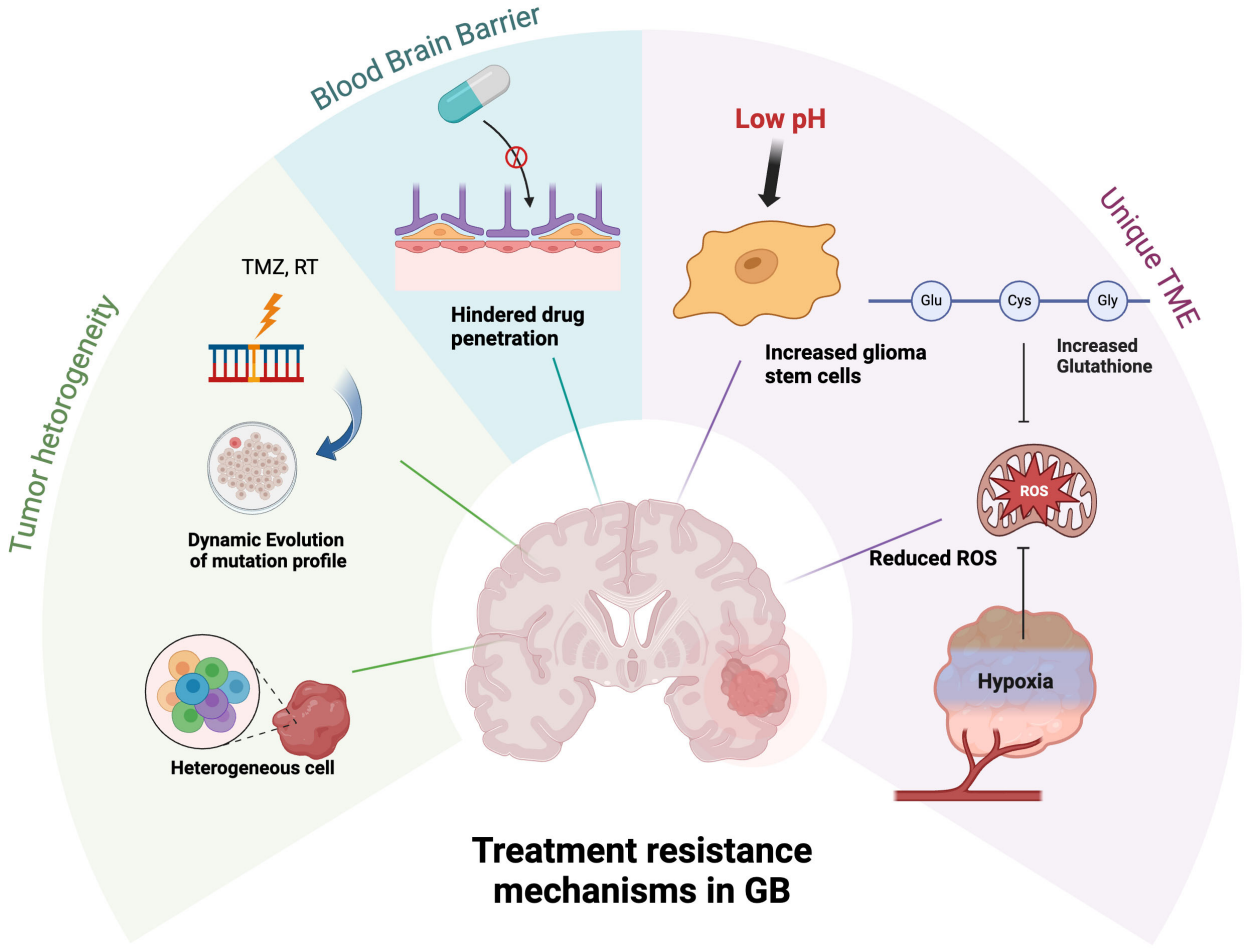
Treatment resistance mechanisms of GB. The tumor cells continuously evolve and diversify the tumor’s genetic profile. Targeted treatment strategies face additional challenges due to poor drug penetration across the BBB. The unique biochemical environment of GB TME, characterized by low pH, hypoxia, and elevated glutathione levels, promotes the proliferation of glioma stem cells and reduces ROS, thereby diminishing the efficacy of anti-neoplastic therapies.

Treatment of GB remains a formidable challenge with limited treatment options, particularly in recurrent settings. Many past attempts to employ targeted therapy, including clinical trials selecting patients whose tumors possess the actionable mutation of interest, have not resulted in substantial clinical benefits. This is largely due to the unique biological and clinical characteristics of GB, including dynamic evaluation of the tumor, its highly heterogeneous tumor microenvironment, and poor drug penetration through BBB, as illustrated in **Figure 3**. These obstacles underscore the need for innovative approaches to improve the understanding and monitoring of tumor biology.

Emerging diagnostic tools such as liquid biopsy offer promising, non-invasive methods to monitor GB. Liquid biopsies using ctDNA provide real-time snapshots of the tumor’s genetic landscape, allowing for adaptive treatment strategies that respond to the tumor’s evolving nature. Although ctDNA detection in blood is limited by the BBB, advances in NGS have improved sensitivity. The regulatory role of miRNA sets them apart as particularly promising biomarkers in liquid biopsy. Further validation of the role of plasma miRNA in GB may result in the identification of GB-specific miRNAs that can be used in lieu of surgery for some patients. EVs can traverse an intact BBB and are valuable in identifying tumor molecular signatures and monitoring treatment response with increased sensitivity. There are other uses of plasma from GB patients. A recent study identified unique metabolomic signatures in the plasma of GB patients at diagnosis and recurrence (157). We believe that serial plasma collection from GB patients during their treatment, along with multi-platform profiling at clinically relevant endpoints (*e.g.*, pre-surgery, post-surgery, recurrence, tissue-proven radionecrosis), can further revolutionize this field and be an extremely valuable diagnostic tool for GB patients.

Recent innovative strategies have shown potential in overcoming GB treatment challenges and enhancing therapeutic agents’ delivery and efficacy. NPs can be engineered to cross the BBB and deliver drugs directly to the tumor site, leveraging mechanisms like receptor-mediated transcytosis. Additionally, MRgFUS can transiently disrupt the BBB, allowing therapeutic agents to penetrate the brain more effectively. Combining this with implantable drug delivery systems, which provide sustained and localized release of therapeutic agents directly to the tumor bed following surgical resection, shows promise. Innovations such as hydrogel-based delivery systems and biodegradable polymer devices have demonstrated efficacy in preclinical models, offering prolonged drug release and improved survival outcomes. These technologies collectively enhance drug delivery efficiency, reduce off-target effects, and improve therapeutic efficacy.

In summary, while the treatment of GB has faced significant difficulties, new strategies such as NPs, FUS, implantable drug delivery systems, liquid biopsy, and adaptive trial platforms offer promising solutions. Continued research and clinical trials are essential to fully realize the potential of these innovative therapies, ultimately improving the prognosis for patients with this aggressive and devastating disease.
